# Behavioral deficits, early gliosis, dysmyelination and synaptic dysfunction in a mouse model of mucolipidosis IV

**DOI:** 10.1186/s40478-014-0133-7

**Published:** 2014-09-09

**Authors:** Yulia Grishchuk, Sarmi Sri, Nikita Rudinskiy, Weiyuan Ma, Katherine G. Stember, Matthew W. Cottle, Ellen Sapp, Marian Difiglia, Alona Muzikansky, Rebecca A. Betensky, Andrew M. S. Wong, Brian J. Bacskai, Bradley T. Hyman, Raymond J. Kelleher, Jonathan D. Cooper, Susan A. Slaugenhaupt

**Affiliations:** 1 Center for Human Genetic Research, Massachusetts General Hospital, Harvard Medical School, 185 Cambridge St., Boston, 02114 MA USA; 2 Pediatric Storage Disorders Lab, Department of Neuroscience, Centre for the Cellular Basis of Behavior and King's Health Partners Centre for Neurodegeneration Research, The James Black Centre, The Institute of Psychiatry, King's College London, 125 Coldharbour Lane, London, SE5 9N UK; 3 Department of Neurology, Massachusetts General Hospital, Harvard Medical School, 114 16th Street, Charlestown, 02129 MA USA; 4 Department of Biostatistics, Harvard School of Public Health, 655 Huntington Avenue, Boston, 02115 MA USA

**Keywords:** Mucolipidosis IV, Lysosomal storage disease, Neuropathology, In vivo Ca2+ imaging, Glia

## Abstract

Mucolipidosis IV (MLIV) is caused by mutations in the gene *MCOLN1*. Patients with MLIV have severe neurologic deficits and very little is known about the brain pathology in this lysosomal disease. Using an accurate mouse model of mucolipidosis IV, we observed early behavioral deficits which were accompanied by activation of microglia and astrocytes. The glial activation that persisted during the course of disease was not accompanied by neuronal loss even at the late stage. *In vivo* [Ca^2+^]-imaging revealed no changes in resting [Ca^2+^] levels in *Mcoln1*^*−/−*^ cortical neurons, implying their physiological health. Despite the absence of neuron loss, we observed alterations in synaptic plasticity, as indicated by elevated paired-pulse facilitation and enhanced long-term potentiation. Myelination deficits and severely dysmorphic corpus callosum were present early and resembled white matter pathology in mucolipidosis IV patients. These results indicate the early involvement of glia, and challenge the traditional view of mucolipidosis IV as an overtly neurodegenerative condition.

**Electronic supplementary material:**

The online version of this article (doi:10.1186/s40478-014-0133-7) contains supplementary material, which is available to authorized users.

## Introduction

Mucolipidosis IV (MLIV) is a lysosomal storage disorder (LSD) with autosomal recessive inheritance caused by loss of function of mucolipin-1 (also known as TRPML1). Over 20 different *MCOLN1* gene mutations have been identified in MLIV patients, although the two founder mutations, both leading to complete loss of mRNA and functional protein, account for ~95% of all MLIV alleles and show a carrier frequency of 1:100 in the Ashkenazi Jewish population [1]. The most profound clinical manifestations of MLIV are severe psychomotor retardation during the first year of life and slowly progressing vision loss due to corneal clouding and retinal degeneration resulting in blindness by the second decade of life [2]–[4]. Neuromotor abnormalities include delayed attainment of motor milestones, spasticity, hypotonia, inability to walk independently, ptosis, myopathic facies, drooling, difficulties in chewing and swallowing, and severely impaired fine-motor function. Most patients reach a level of motor, speech and cognitive development of about 15 months, and remain neurologically stable during second and third decade of life [5],[6]. The most striking MRI findings in MLIV include a dysplastic corpus callosum, widespread white matter abnormalities, including abnormal diffusion weighed imaging values: increased mean diffusivity and decreased fractional anisotropy, decreased T2 signal intensities in the thalamus due to increased ferritin deposition, and cerebellar atrophy in older patients [6]–[8]. At the cellular level MLIV results in the formation of abundant electron-dense inclusions composed of lipid-like (lamellated) and polysaccharide-like (granular) material, referred to as "compound bodies" [9],[10].

TRPML1 functions as a non-specific cation channel [11]–[13]. Previous studies by our group and others showed TRPML1 localization to late endosomes and lysosomes (LEL) [14]–[17], and its involvement in lipid trafficking [18],[19]; Ca^2+^-dependent LEL fission-fusion events [19]; reformation of lysosomes from endosome-lysosome hybrids [17],[20] and autolysosomes [21],[22]; and lysosomal exocytosis [23],[24]. However, the endogenous localization and function of TRPML1 in both neurons and glial cells in the brain have yet to be defined.

A mouse model of MLIV created in our laboratory was shown to accurately recapitulate the key features of the human disease [25]. At birth, the *Mcoln1* knock-out mice display no overt phenotypes; limb weakening is observed at the age of 3 months and progresses to total hind limb paralysis and death by approximately 8 months. Ultrastructural analysis revealed presence of the storage inclusions in neurons and all types of glial cells. Inclusions resemble the "compound bodies" reported in MLIV patients and are detectable in the embryonic mouse brain. Histological analysis of end-stage brains revealed accumulation of gangliosides, cholesterol and P62/SQSTM1, as well as glial activation and reduced myelination [26]. The mechanisms that link loss of TRPML1 with brain pathology and the devastating neurological symptoms of MLIV remain unclear, and there is currently no treatment for the disease. Therefore, study of the MLIV mouse model is an essential step toward understanding disease pathogenesis and for testing potential therapies. Here we investigate the onset and progression of neurological phenotypes in *Mcoln1*^*−/−*^ mice using behavioral testing, systematic volumetric and neuropathologic analysis of post-mortem brain tissue, *in vivo* multiphoton imaging of resting [Ca^2+^] concentration and electrophysiology. Our data indicate an early and profound involvement of glial cells in pathogenesis of MLIV, a disease traditionally viewed as being "neuronal", and provide new clues to the development of therapies for this devastating disorder.

## Materials and methods

### Animals

*Mcoln1* knock-out mice were maintained and genotyped as previously described [25]. The *Mcoln1*^*+/−*^ breeders for this study were obtained by backcrossing onto a C57Bl6N background for more than 10 generations. *Mcoln1*^*+/+*^ littermates were used as controls. Experiments were performed according to the institutional and US National Institute of Health guidelines and approved by the Massachusetts General Hospital Institutional Animal Care and Use Committee.

### Open field testing

Open field testing was performed on naive male mice at one (n = 13, wild type; n = 9, *Mcoln1*^*−/−*^) and two (n = 17, wild type; n = 18, *Mcoln1*^*−/−*^) months of age under regular light conditions. Each mouse was placed in the center of a 27 × 27 cm^2^ Plexiglas arena, and the horizontal and vertical activity were recorded by the Activity Monitor program (Med Associates). Data were analyzed during the first 10 mins in the arena. Zone analysis was performed to measure movements/time spent in the central (8 × 8 cm^2^) versus peripheral (residual) zone of the arena. Data were analyzed by two-way ANOVA (genotype × age) followed by Bonferroni post-test.

### Stereological analysis and immunohistochemistry

To obtain brain tissue for histological examination two (n = 6 per genotype), three (n = 3, WT; n = 4, *Mcoln1*^*−/−*^) and seven month-old *Mcoln1*^*−/−*^ and control mice (n = 4 per genotype) were transcardially perfused under isoflurane anesthesia with ice-cold phosphate buffered saline (PBS) followed by 4% paraformaldehyde in PBS. Brains were postfixed in 4% paraformaldehyde in PBS for 24 hours, washed with PBS, cryoprotected in 30% sucrose in PBS overnight, frozen in isopentane and stored at −80°C. Brains were bisected along the midline and one hemisphere was examined histologically. 40 μm coronal sections were cut using a Microm freezing microtome and collected into 96 well plates containing TBSAF (TBS, 30% ethylene glycol, 15% sucrose, 0.05% sodium azide). These sections were stored at 4°C prior to any staining procedures. For Nissl staining a one-in-six series of sections were mounted onto chrome gelatine coated slides, incubated in 0.05% cresyl violet stain and 0.05% acetic acid at 60°C for 30 mins before being dehydrated in ascending concentration of industrial methylated spirits (IMS) followed by xylene. The sections were then coverslipped using DPX mountant.

### Quantitative histological measurements

Nissl stained sections were used to obtain cortical thickness measurements using *StereoInvestigator* software. All analyses were carried out using ×2.5 objective on a Zeiss, Axioskop2 MOT microscope (Carl Zeiss Ltd, Welwyn Garden City, UK). Briefly, within each region three consecutive sections were chosen and 10 perpendicular lines were drawn from the pial surface to the white matter.

Unbiased Cavalieri estimates of regional volume were obtained using *StereoInvestigator* software. Briefly, for each of the regions a sampling grid size of 150 μm was superimposed on every one-in-twelfth Nissl stained section (cortex and hippocampus) or every one-in-six Nissl stained section (thalamus and striatum) and the number of points that fell within this region was recorded to provide an unbiased Cavalieri estimate of regional volume (μm^3^) for each brain. All measurements were carried out at ×2.5 objective on the Olympus BX50 microscope (Olympus Microscopes, South-on-Sea, UK).

Unbiased optical fractionator estimates for the number of Nissl stained neurons within the VPL-VPM, laminae IV and V of the S1BF, DLG and red nucleus were obtained using the *StereoInvestigator* program. A grid size of 175 μm × 175 μm was used for the thalamus, 225 μm × 225 μm was used for laminae IV and V of the S1BF, and 125 μm × 125 μm was used for the DLG and red nucleus. A counting frame of 68 cm × 38 cm was used for all regions and all measurements were carried out at ×100 objective on a Zeiss, Axioskop2 MOT microscope (Carl Zeiss Ltd, Welwyn Garden City, UK). The mean coefficient of error (CE) for optical fractionator estimates of neuron number were all between 0.05 and 0.1. All analyses were performed blind to genotype, which was only revealed once these analyses were complete. At this point the mean value for each parameter was calculated for both *Mcoln1-* deficient and +/+mice. Differences between genotypes were compared statistically using Student's t-tests with a P-value of ≤0.05 considered as statistically significant.

### Immunostaining

Immunostaining was performed as described [27]. The primary polyclonal rat anti-CD68 (Serotec, 1:2000) and polyclonal rabbit anti-GFAP (DAKO, 1:4000) antibodies were used. For myelin FluoroMyelin™ Green fluorescent stain (Molecular Probes) was used according to the manufacturer protocol.

### Thresholding image analysis

Three consecutive GFAP- and CD68- immunostained sections were chosen for each of the regions being observed and 30 non-overlapping images were captured at 40× objective by a live video camera (JVC, 3CCD, KY-F55B) mounted on Zeiss Axioplan microscope (West Germany). The lamp intensity, video camera setup, and calibration were kept constant when capturing all the images. These images were then analysed on *ImageProPlus* software with an appropriate threshold selected to distinguish the foreground immunostaining above the background for each of the age groups being observed. The data obtained from the thresholding analysis was plotted graphically as a mean percentage area of immunoreactivity per image.

### In vivo imaging of Ca2+ concentrations in cortical neurons

To deliver the genetically encoded calcium indicator, craniotomies were performed on *Mcoln1*^*−/−*^ (n = 5) and control mice (n = 3) of 2 months and 1 week of age. A 5-mm diameter skull flap centered over the right primary somatosensory cortex was removed and 2 μl of AAV2/8 viral vector (5.6 × 10^12^ viral genomes/ml) encoding YC3.60 under hybrid cytomegalovirus (CMV) immediate-early enhancer/chicken β-actin promoter/exon1/intron [28] were injected into the open brain close to the center of the craniotomy at a depth of about 0.5 mm and at a speed of 0.2 μl/min. The brain surface (still covered by intact dura matter) was kept moist with Ringer's solution at all times. Following the virus injection, the craniotomy was sealed with a glass coverslip and cemented with dental acrylic. Appropriate anesthetic and analgesic regimes were followed before, during and after the surgery. 3 weeks after the surgery, when the mice reached 3 months of age, they were anesthetized (isoflurane in balanced oxygen: 4% for 5 min induction, then maintained at 1.2%), head fixed and imaged on a multiphoton microscope. Our imaging setup was described earlier [29]. The excitation laser was tuned to 860 nm and the output power before the objective was set to 30 mW. Emitted light was collected in three channels: 460 – 500 nm (cyan channel (C), CFP fluorescence), 530 – 560 nm (yellow channel (Y), YFP fluorescence) and 575 – 630 nm (red channel (R), autofluorescence from storage material). With these settings, autofluorescent storage material yielded equal signals in cyan, yellow and red channels. Z-stacks were acquired with the resolution of 0.5 μm/voxel in X-Y dimension and the Z-step of 3 μm for imaging of neuronal cell bodies in the cortical layer II/III (512 × 512 × 270 μm stack) and with the resolution of 0.25 × 0.25 × 2 μm/voxel for imaging of dendrites in the cortical layer I (512 × 512 × 60 μm stack). Imaging settings were kept constant across mice.

Image processing was performed using the Fiji package of NIH ImageJ software (fiji.sc; rsbweb.nih.gov/ij) and MATLAB (MathWorks). Ratio of YFP to CFP signals representative of intracellular calcium concentration was calculated after subtracting the signal from background-subtracted red channel:$R=\frac{Y-R}{C-R}$. Regions of interest (ROIs) outlining cell bodies and dendrites were selected on the raw images and applied to the ratio images and to the red channel to estimate the amount of autofluorescent storage. The values for individual cells and dendrites were calculated as mean ratio in ROIs. All statistical tests were performed on *R* values. To convert *R* values to [Ca^2+^] for data presentation and interpretation, the following formula was used:Ca2+=Kd′R−RminRmax−R1n. Values for$K_{d}^{-}$(277 nM) and the Hill coefficient *n* (1.1) were described previously [28]. *R*_*min*_ (0.67) and *R*_*max*_ (2.55) were measured experimentally. To create pseudocolored images, ratio images coded with "Rainbow RGB" lookup table were converted to the RGB space weighted by intensity.

The normality of datasets of YC3.60 ratio values was tested using Kolmogorov-Smirnov method. Since the distributions were found to be non-normal, they were compared between genotypes using the Wilcoxon rank-sum test with correction for clustering of values within individual mice [30]. The correlation of YC3.60 ratio values with the amount of cytosolic storage material was performed using a mixed effects model, with random slope effects for mouse, and using mouse-specific standardized transformations of YC3.60 ratio and autofluorescence values.

### Electrophysiology

Transverse hippocampal slices were prepared from 7 month-old *Mcoln1*^*−/−*^ and control male mice (n = 6 per genotype) and field recordings were performed as described [31]. Data were normalized to the baseline response and are presented as group means ± SE. One-way ANOVA and Student's t-test were used to determine statistically significant differences. For all experiments the experimenter was blind to genotype.

### Electron microscopy

Seven month-old *Mcoln1*^*−/−*^ and control mice (n = 4 per genotype) were anesthetized with isoflurane and transcardially perfused with PBS followed by 2% paraformaldehyde/2% glutaraldehyde in PBS, brains were removed and 50 μm coronal serial vibratome sections were processed for EM as previously described [32]. 35 micrographs per sample from stratum radiatum per mouse were obtained on a JEOL JEM-1011 transmission electron microscope at final magnification of ×25,000. Only asymmetric macular (non-perforated) synapses that were fully present on the micrograph were included in the analysis. Digital image analysis was performed using ImageJ (NIH). The post-synaptic density length was measured. Analysis of mitochondrion volume was performed as described [33]. For myelin sheath thickness measurements two perpendicular lines were drawn through the center of each myelinated axon and four measurements of the thickness were taken at each intersection with the myelin sheath with the mean value of four measurements calculated. The normality of all data sets was tested using the Kolmogorov-Smirnov method. Non-parametric data are presented as median values with interquartile ranges. An extended Wilcoxon rank-sum test that accommodates clustered data was used for comparisons between control and *Mcoln1*^*−/−*^ groups for non-normally distributed data. Statistical significance threshold was set at p < 0.05.

## Results

### Early motor and cognitive deficits in Mcoln1−/−mice

**Figure 1:**
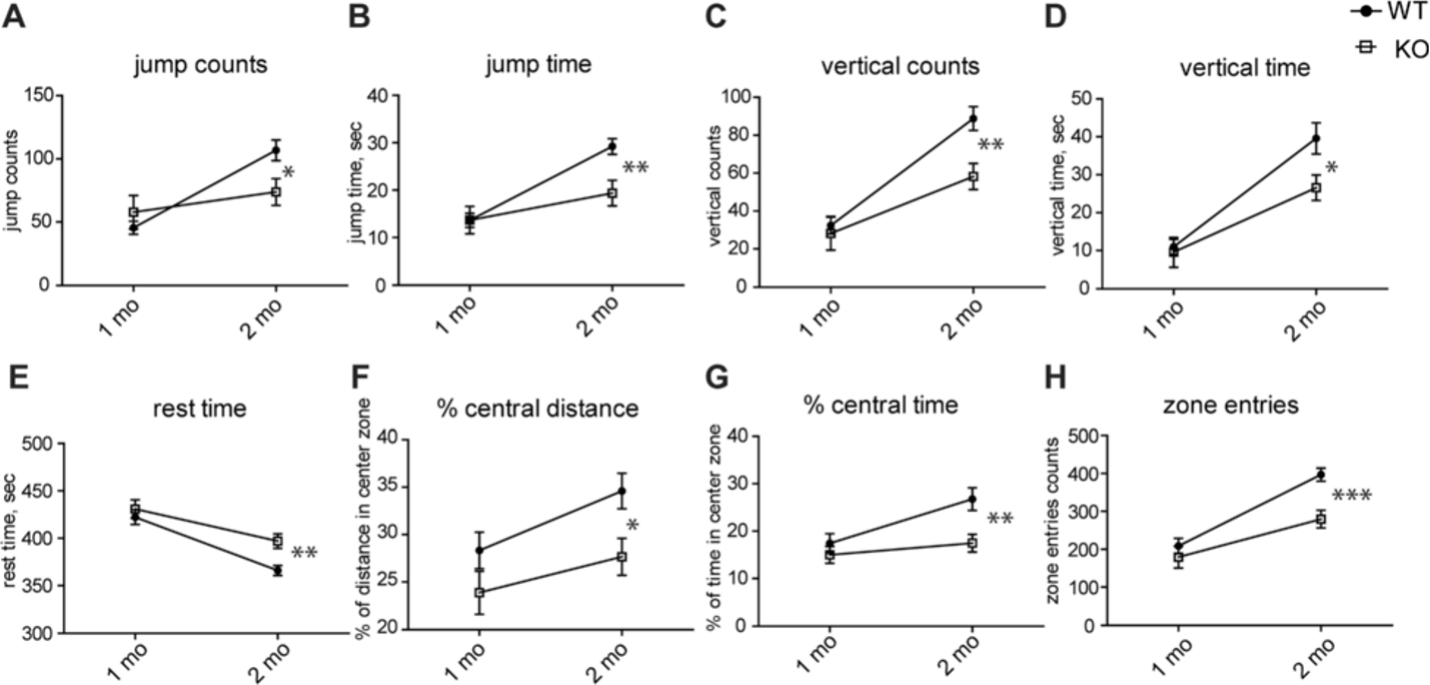
**Motor and exploratory activity in the open field test is decreased in the two month-old** ***Mcoln1*** ^***−/−***^ **mice.** Data presented as mean values ± SEM for 8 wild-type (WT) and 13 *Mcoln1* ^−/−^ (KO) one month-old and 17 wild-type and 18 *Mcoln1* ^−/−^ two month-old mice. Each mouse, at either one or two months of age, was tested once in the open field and spontaneous activity during 10 min of exploration in the open-field arena was recorded. Two-way ANOVA analysis revealed: significant interaction between age and genotype in jump counts (F _genotype × age_ = 6.96, p = 0.0271) **(A)** and jump time (F _genotype × age_ = 5.36, p = 0.0409) **(B)**; significant effects of age and genotype on vertical activity, expressed as rearing (F _genotype_ = 6.16, p = 0.0155; F _age_ = 38.09, p < 0.0001) **(C)**, and vertical time (F _genotype_ = 3.87, p > 0.05; F _age_ = 38.7, p < 0.0001) **(D)**, resting time (F _genotype_ = 6.91, p = 0.0128; F _age_ = 35.75, p < 0.0001) **(E)** and exploratory activity, expressed by percentage of central track length (F _genotype_ = 11.15, p = 0.0087; F _age_ = 8.69, p = 0.0197) **(F)**, percentage of time spent in the center (F _genotype_ = 9.99, p = 0.0111; F _age_ = 10.12, p = 0.0106) **(G)** and the number of central zone entries (F _genotype × age_ = 5.36, p = 0.0409) **(H)**. Asterisks indicate significant difference between genotypes at given time-points as determined by post-hoc t-test with Bonferroni correction, p* - <0.05, ** - <0.01, *** - <0.001.

To determine if *Mcoln1*^*−/−*^ mice display early locomotor and behavioral deficits, spontaneous activity of one- and two-month-old naïve *Mcoln1*^*−/−*^ mice and wild-type littermates was tested in the open field arena. We found a significant decline in both jumping and vertical activity and an increase in the resting time in *Mcoln1*^*−/−*^ mice at two months of age (Figure 1A-E), which indicates the early onset of motor deficits. Analysis of activity within the central zone revealed that the number of central zone entries, percentage of central track length and percentage of time spent in the center were significantly lower in *Mcoln1*^*−/−*^ mice compared to wild-type controls at 2 months of age (Figure 1F-H) indicating an early decline in the exploratory activity associated with loss of *Mcoln1*.

### Absence of brain atrophy in Mcoln1−/−brain

Neuropathology in models of lysosomal storage disorders is typically characterized by atrophy and neuronal loss within specific brain regions. To assess the progression of anatomical changes in the *Mcoln1*^*−/−*^ brain, we performed unbiased stereological measurements of the volume of four brain regions (cortex, hippocampus, thalamus and striatum) in two, three and seven month-old mice. We observed no atrophy of any of these brain regions at any of these three time points (Additional file 1: Figure S1A). Next we assessed whether more subtle effects were evident within the cortex of these mice by obtaining cortical thickness measurements from the somatosensory barrelfield cortex (S1BF), primary motor cortex (M1), lateral entorhinal cortex (LEnt) and primary visual cortex (V1) at two, three and seven months. No significant changes in cortical thickness were detected between *Mcoln1*^*−/−*^ and control mice at any age (Additional file 1: Figure S1B), suggesting that none of the examined regions of cortex undergoes overt neurodegeneration during the course of this disease.

### Profound early activation of microglia and astrocytes in Mcoln1−/−mice

**Figure 2:**
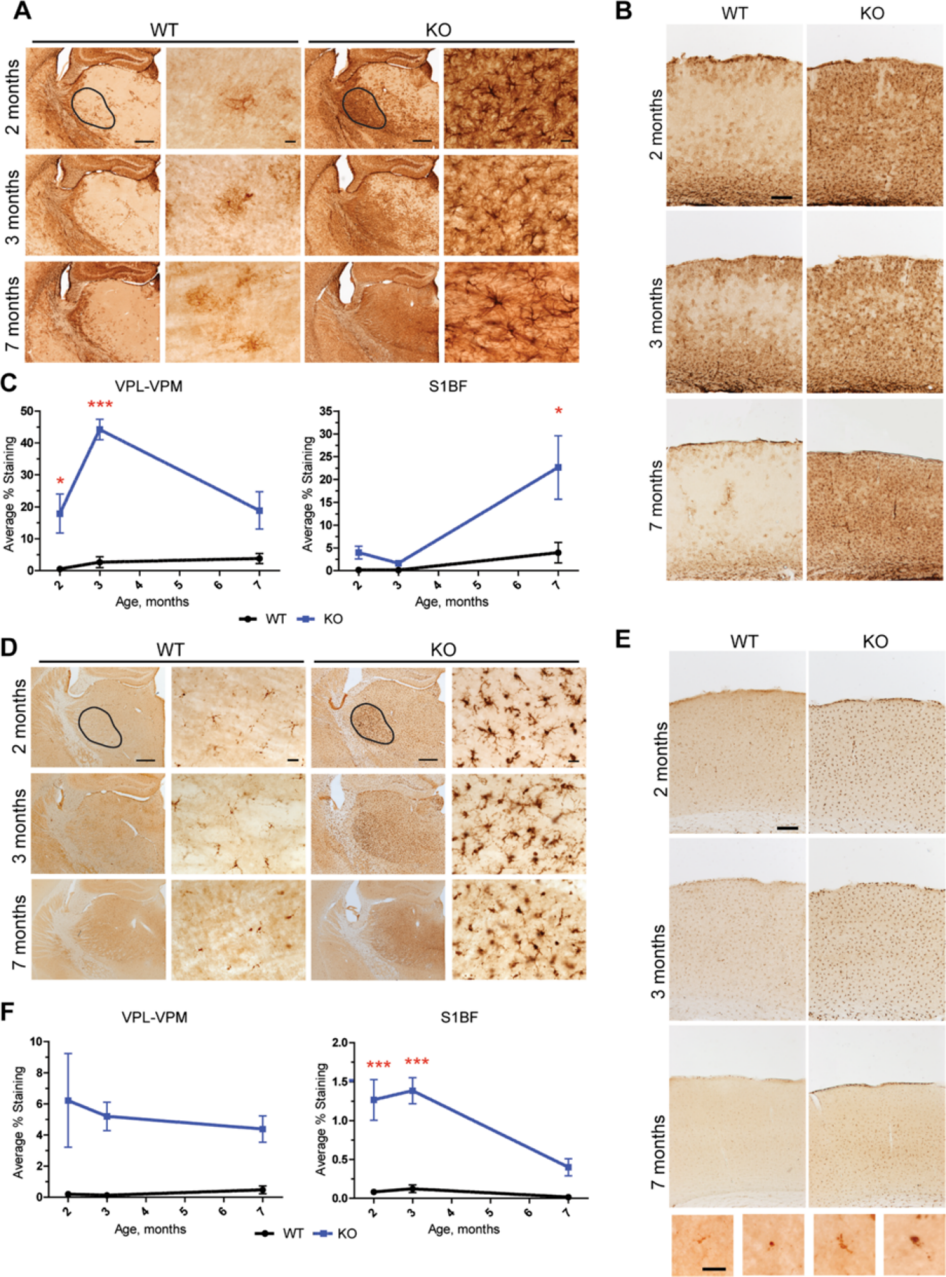
**Reactive astrocytosis and activation of microglia in** ***Mcoln1*** ^***−/−***^ **mice.** Representative images of GFAP immunostaning in wild-type (WT) and *Mcoln1* ^−/−^ (KO) littermates at two, three and seven months of age showing increased immunoreactivity in KO in thalamus **(A)** or somatosensory barrelfield cortex **(B)**. Scale bar is equal to 500 μm in low magnification images, 50 μm in higher magnification images of thalamic area and 150 μm in the images of cortex. Black selection area shows an example of VPL/VPM area of thalamus used for thresholding analysis, represented in **(C)**. **(C)**. Thresholding analysis of GFAP staining intensity performed for VPL-VPM region of thalamus and S1BF cortex in wild-type (WT) and *Mcoln1* ^−/−^ (KO) littermates at two (*n* = 6 per genotype), three (*n* (WT) = 3; *n* (KO) = 4) and seven months of age (*n* = 4 per genotype). Data presented as mean values ± SEM. Two-way ANOVA revealed significant effect of age and genotype. Post-hoc t-test with Bonferroni correction: * - p < 0.05; *** - p < 0.001. **(D)**, **(E)**. Representative images of CD68 immunostaning in wild-type (WT) and *Mcoln1* ^−/−^ (KO) littermates at two, three and seven months of age showing increased immunoreactivity in KO in thalamus **(D)** or somatosensory barrelfield cortex **(E)**. Scale bars as in **(B)**. Black selection area shows an example of VPL-VPM area of thalamus used for thresholding analysis, represented in **(F)**. **(F)**. Thresholding analysis of CD68 staining intensity in VPL/VPM region of thalamus and S1BF cortex in wild-type (WT) and *Mcoln1* ^−/−^ (KO) littermates at two (*n* = 6 per genotype), three (*n* (WT) = 3; *n* (KO) = 4) and seven months of age (*n* = 4 per genotype group). Data presented as mean values ± SEM and analyzed by two-way ANOVA and post-hoc t-test with Bonferroni correction: *** - p < 0.001.

Since pronounced activation of microglia and astrocytes has been reported in the single available autopsy report from an MLIV patient [9], and in end-stage *Mcoln1*^*−/−*^ mice [26], we evaluated the onset and progression of these phenotypes in brain tissue from two, three and seven month-old *Mcoln1*^*−/−*^ mice. Immunostaining for the astrocyte marker GFAP revealed marked reactive astrocytosis in *Mcoln1*^*−/−*^ brains as early as two months of age. At this time we observed an intense GFAP immunoreactivity in the somatosensory barrelfield cortex, primary motor cortex and hypothalamus, with the most prominent staining intensity in the VPL/VPM region of thalamus (Figure 2A, B). Astrocytosis in *Mcoln1*^*−/−*^ mice progressed with age, and by the age of seven months intensely stained GFAP-positive cells were present throughout the entire area of thalamus and hypothalamus. These GFAP-positive astrocytes within the *Mcoln1*^*−/−*^ brain appeared hypertrophied with enlarged soma and thickened processes. However, astrocytosis appeared to progress at different rates between brain regions. For example, staining for GFAP was more pronounced in VPL/VPM at earlier stages of disease, and only subsequently became more evident in S1BF, the target cortical region for this somatosensory relay nucleus of the thalamus (Figure 2C). Thresholding image analysis in the VPL/VPM area of thalamus revealed a significant effect of age and genotype on GFAP staining intensity (F _genotype × age_ = 5.67; p < 0.0107) with increased staining in *Mcoln1*^*−/−*^ mice at two and three months of age (post-hoc t-test with Bonferroni correction: p _2 months_ < 0.05; p _3 months_ < 0.001; p _7 months_ > 0.05). GFAP staining intensity in S1BF cortex was also significantly affected by genotype and age (F _genotype × age_ =5.54; p = 0.0117), however post hoc test revealed significant increase in GFAP in *Mcoln1*^*−/−*^ mice only at the age of seven months (p < 0.01).

To examine the microglial response, brain tissue from two, three and seven month-old *Mcoln1*^*−/−*^ mice and wild-type littermates was immunostained for the microglia/macrophage marker CD68. Compared to the wild-type controls, the *Mcoln1*^*−/−*^ mice displayed pronounced activation of microglia early in disease progression, which was evident at two months of age, especially within the VPL/VPM (Figure 2D), throughout the cortex (Figure 2E), hippocampus, DLG and red nucleus (not shown). However, microglial response became less pronounced and widespread in *Mcoln1*^*−/−*^ mice at seven months of age. Microglia in control mice were faintly stained and exhibited a ramified morphology with a small cell body, whereas in the *Mcoln1*^*−/−*^ mice they were much more intensely stained and displayed more rounded and enlarged cell bodies, with short thickened processes characteristic of activated microglia or brain macrophages (Figure 2D, E). Thresholding image analysis in the VPL/VPM region of thalamus followed by two-way ANOVA test showed a significant effect of genotype (F _genotype_ = 11.54; p = 0.0027), and no effect of age (F _age_ = 0.098; p = 0.91) on CD68 staining intensity (Figure 2F). Analysis of CD68 staining in the S1BF region of the cortex revealed significant effect of genotype and age (F _genotype × age_ = 4.1; p = 0.03), with a significant increase in CD68 staining intensity at two (p < 0.001) and three (p < 0.001), but not at seven months (Figure 2E, F), when activated microglia appeared to be more localized to lamina IV. Similar changes in microglia morphology and increased staining intensity were observed with the microglial marker Iba1 (Additional file 2: Figure S2).

Ultrastructural analysis of the brain tissue (CA1 stratum radiatum of hippocampus) at seven months revealed accumulation of electron-dense storage bodies in *Mcoln1*^*−/−*^ astrocytes similar to those described previously in the cortex [25] (Additional file 3: Figure S3A). We also observed the formation of large aggregates of electron-dense organelles (or clumps). Most often they were engulfed by the plasma membrane which lacks synaptic contacts and, based on cell morphology [34], most likely belongs to microglia/macrophages (Additional file 3: Figure S3B).

### Normal resting calcium concentrations in the somatosensory cortex of Mcoln1−/−mice

**Figure 3:**
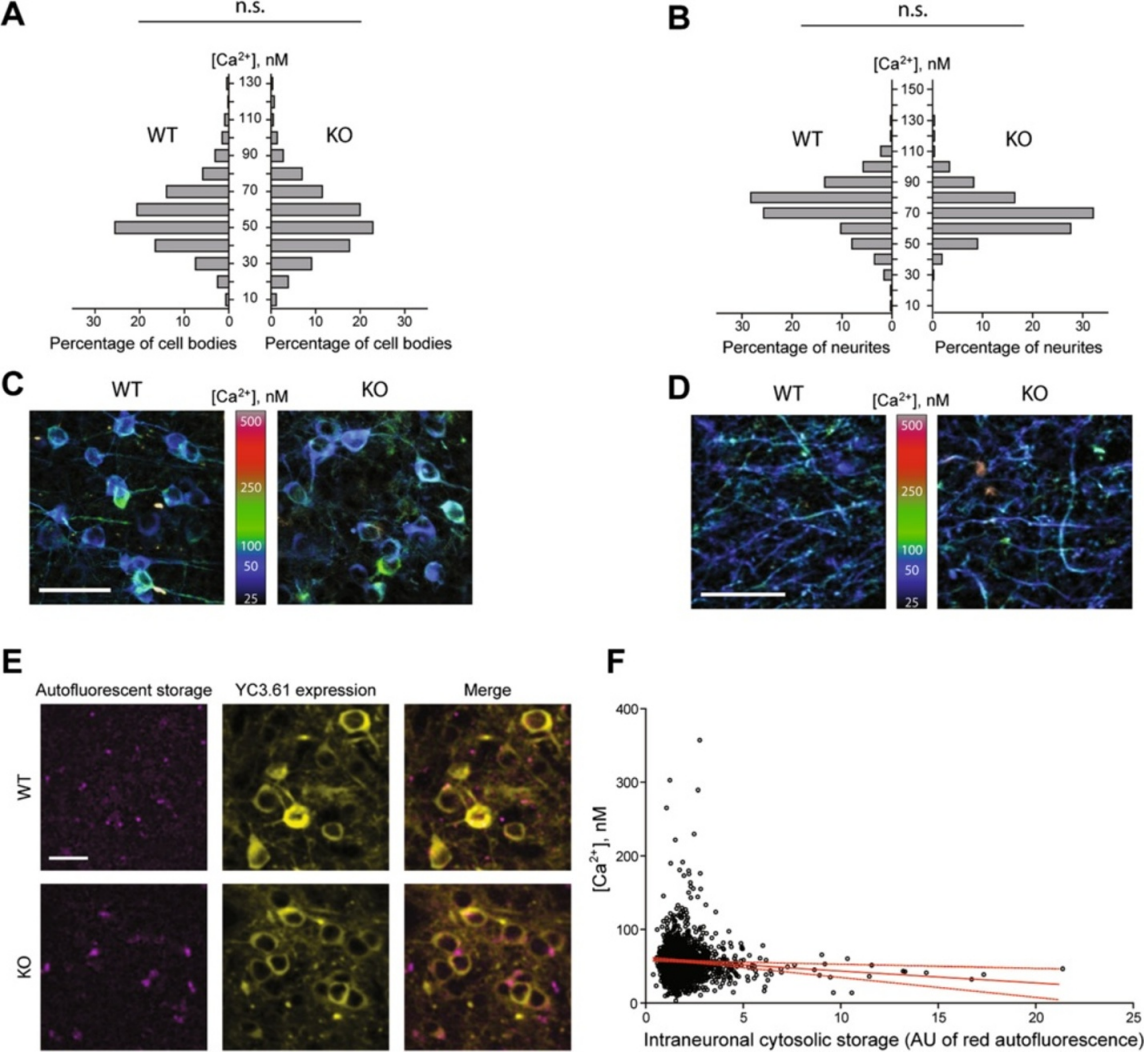
**Intracellular resting [Ca** ^**2+**^ **] is normal in the cortical neurons of** ***Mcoln1*** ^***−/−***^ **mice.** Frequency distributions of resting intracellular [Ca^2+^] levels in the neuronal cell bodies in the primary somatosensory cortical layer II/III **(A)** and in the apical dendrites of these neurons in the layer I **(B)**. N.s. – no significant difference in distributions between WT and KO mice; p = 0.49 (cell bodies), p = 0.86 (dendrites); *n* = 536 dendrites and 1825 neurons from 5 mice (KO), *n* = 312 dendrites and 888 neurons from 3 mice (WT), assessed using clustered rank-sum test [30]. Maximum intensity Z-projections of representative 75-μm thick multiphoton image stacks demonstrating YC3.60 expression in neuronal cell bodies in layer II/III **(C)** and in apical dendrites in layer I **(D)**. The projections were pseudocolored based on measured [Ca^2+^] levels. Scale bar = 50 μm **(C)**, 20 μm **(D)**. **(E)**. Representative multiphoton sections from layer II/III of somatosensory cortex of *Mcoln1* ^*−/−*^ and control mice demonstrating intracellular autofluorescent storage material in the YC3.60-expressing neurons. YC3.60 expression is displayed as the sum of signals in the cyan and yellow channels. Scale bar = 50 μm. **(F)**. Plot of intracellular [Ca^2+^] in the neuronal cell bodies in KO mice versus the amount of autofluorescent cytosolic storage material in the same cells. Solid red line shows linear regression, dotted red line – 95% confidence interval. Correlation coefficient = −0.074, p = 0.04. AU = arbitrary units of fluorescence. *n* = 1825 neurons in 5 mice.

To assess if loss of TRPML1 and the early activation of glia resulted in sub-lethal neurotoxicity and impaired calcium homeostasis, we performed *in vivo* measurements of intracellular calcium concentrations in cortical neurons of *Mcoln1*^*−/−*^ and wild-type mice expressing the genetically-encoded ratiometric calcium sensor YC3.61 [28],[35]. An adeno-associated viral vector (AAV 2/1) was used to deliver YC3.61 to layer II/III neurons of the somatosensory cortex, a region where pronounced astrocytosis and microglial activation is present (Figure 2). Resting [Ca^2+^] was determined in the individual cell bodies and dendrites by *in vivo* multiphoton imaging of the injected area through implanted cranial window [28] in anesthetized mice. Analysis of resting calcium concentration in three month-old mice revealed no differences in either neuronal somata in layers II/III (Figure 3A, C) (3 WT mice, 888 cells; 5 KO mice 1875 cells analyzed) or neuropil in layer I (Figure 3B, D) (3 WT mice, 312 neurites; 5 KO mice 536 neurites analyzed). Resting [Ca^2+^] levels in the dendrites of both KO mice and control littermates were in good concordance with the previously reported values for wild-type mice [28]. Additionally, we observed large autofluorescent storage aggregates in the cytosol of *Mcoln1*^*−/−*^ neurons (Figure 3E) which, however, had very little effect on the levels of intraneuronal resting [Ca^2+^] (R = –0.074, p = 0.04), and even the cells with the highest amount of storage material had physiologically normal [Ca^2+^] levels (Figure 3E, F). These data show that the loss of TRPML1 does not result in neuroexcitotoxicity and provides evidence of physiological health of *Mcoln1*^*−/−*^ neurons at three months of age despite profound activation of microglia and astrocytes in the somatosensory cortex at this age.

### Activation of microglia and astrocytes is not accompanied by neuronal loss in Mcoln1−/−mice

Activation of microglia has been linked to neuron loss in many neurodegenerative diseases [36]. To assess if chronic activation of microglia was associated with neuronal loss in *Mcoln1*^*−/−*^ mice later in disease progression, unbiased optical fractionator neuron counts were obtained in the brain areas with the most prominent microgliosis, i.e. the VPL/VPM region of thalamus, laminae IV and V of the somatosensory barrelfield cortex, the CA1 and CA3 subfields of the hippocampus, the DLG and red nucleus, in seven month-old mice (Additional file 4: Figure S4). We observed no significant differences in neuron number between wild type and *Mcoln1*^*−/−*^ mice in any of the examined regions, suggesting that activation of microglia was not caused by or resulted in neuronal death in the *Mcoln1*^*−/−*^ brain.

### Abnormal synaptic plasticity in Mcoln1−/−mice

**Figure 4:**
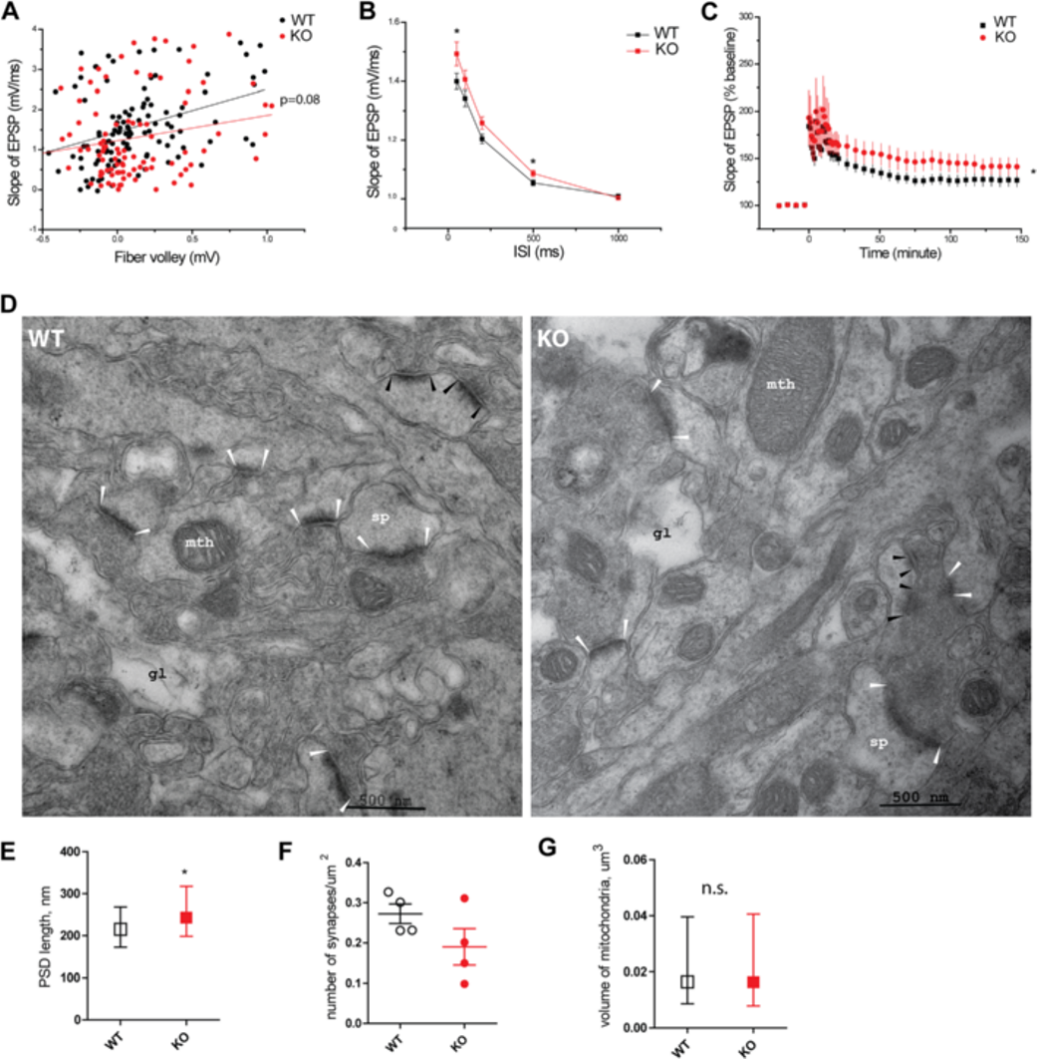
**Synaptic plasticity and altered synapse morphology in** ***Mcoln1*** ^***−/−***^ **mice. (A)**. Basal synaptic transmission: the synaptic input–output curve shows fEPSP slopes as a function of fiber-volley amplitudes for wild-type (*n* = 24 slices, 6 mice) and *Mcoln1* ^*−/−*^ (*n* = 26 slices, 6 mice). The lines represent linear regression of the data; p = 0.08. **(B)**. The paired-pulse facilitation (PPF) is enhanced in *Mcoln1* ^*−/−*^ mice. The facilitation ratio is shown as a function of interpulse interval (ISI) for wild-type (*n* = 29 slices, 6 mice) and *Mcoln1* ^*−/−*^ (*n* = 31 slices, 6 mice) slices. *, p < 0.05. **(C)**. Increased LTP in *Mcoln1* ^*−/−*^ mice. LTP was induced in control (*n* = 18 slices, 6 mice) and *Mcoln1* ^*−/−*^ slices (n = 16 slices, 6 mice) with 4 tetanic trains (100 Hz, 1 s) separated by 5 min each. *, p < 0.05. fEPSP recordings were filtered at 1 kHz, digitized at 20 kHz, and analyzed using pClamp10 (Axon Instruments). **(D)**. Electron micrographs of CA1 stratum radiatum: white arrowheads indicate examples of PSDs in macular excitatory (glutamatergic) synapses included in the analysisin **(E)**; black arrows show post-synaptic densities of active zones in the perforated excitatory synapses omitted from the analysis. Mth, mitochondria; gl, process of a glial cell; sp – dendritic spine. **(E)**. PSD length quantification in 4 control and 4 *Mcoln1* ^*−/−*^ mice, total number of 433 control and 402 *Mcoln1* ^*−/−*^ synapses. Data presented as median values with interquartile range, *, p (clustered rank-sum test) <0.05. **(F)**. Synapse density quantification. Each data point represents the average number of synapses per μm^2^ per mouse; line bars represent average value per genotype group and SEM; p (Student's t-test) >0.05. **(G)**. Volume of mitochondria: total number of 1262 control and 1196 *Mcoln1* ^*−/−*^ mitochondria measured (n = 4 mice per genotype); n.s., p (clustered rank-sum test) > 0.05.

Since our data show no overt neuronal loss in the *Mcoln1*^*−/−*^ brain, we next examined the consequence of loss of TRPML1 on neuronal function, which might explain the severe motor and cognitive decline in MLIV. To assess synaptic function, we performed field recordings in the Schaeffer collateral pathway of acute hippocampal slices from seven month-old control and *Mcoln1*^*−/−*^ mice (n = 6). First, we examined basal synaptic transmission by measuring the synaptic input–output relationship, plotting the slope of the field excitatory postsynaptic potential (fEPSP) as a function of the presynaptic fiber volley amplitude at varying stimulus intensities. Our results revealed a non-significant trend toward decreased synaptic input–output coupling in the *Mcoln1*^*−/−*^ relative to control mice (p = 0.08) (Figure 4A). Second, we analyzed paired-pulse facilitation (PPF), a short-term form of presynaptic plasticity in which paired stimulation with a short inter-stimulus interval elicits a transient enhancement in neurotransmitter release. We observed significantly elevated PPF in the *Mcoln1*^*−/−*^ hippocampus at the shortest inter-stimulus intervals (ISI) (Figure 4B), implying that TRPML1 deficiency may affect neurotransmitter release. We also examined the impact of TRPML1 loss on synaptic plasticity by analyzing long-term potentiation (LTP), a long-lasting enhancement of synaptic strength induced by patterned high-frequency stimulation, which is thought to model synaptic modifications underlying learning and memory. Experiments in which LTP was induced by multiple trains of tetanic stimulation revealed that loss of TRPML1 led to significantly enhanced LTP (Figure 4C).

### Alterations in synapse morphology in Mcoln1−/−mice

To determine if there were morphological changes associated with altered synaptic plasticity in *Mcoln1*^*−/−*^ mice, we performed electron microscopic analysis of synaptic terminals in the CA1 stratum radiatum of 7 month-old mice. We observed elongated post-synaptic densities (PSD) in excitatory (asymmetric) synapses (p = 0.024) in *Mcoln1*^*−/−*^ mice compared to control littermates (Figure 4D, E). Quantification of the asymmetric synapse density in CA1 stratum radiatum showed a trend towards a decrease in *Mcoln1*^*−/−*^ mice, but this did not reach statistical significance due to high variability (Figure 4F). Since axonal pathology is often accompanied by mitochondrial stress, we measured volume of mitochondria in neuropil of CA1 stratum radiatum in *Mcoln1*^*−/−*^ and control mice, but found no difference (Figure 4G).

### Loss of Mcoln1 results in the early defects of myelination

**Figure 5:**
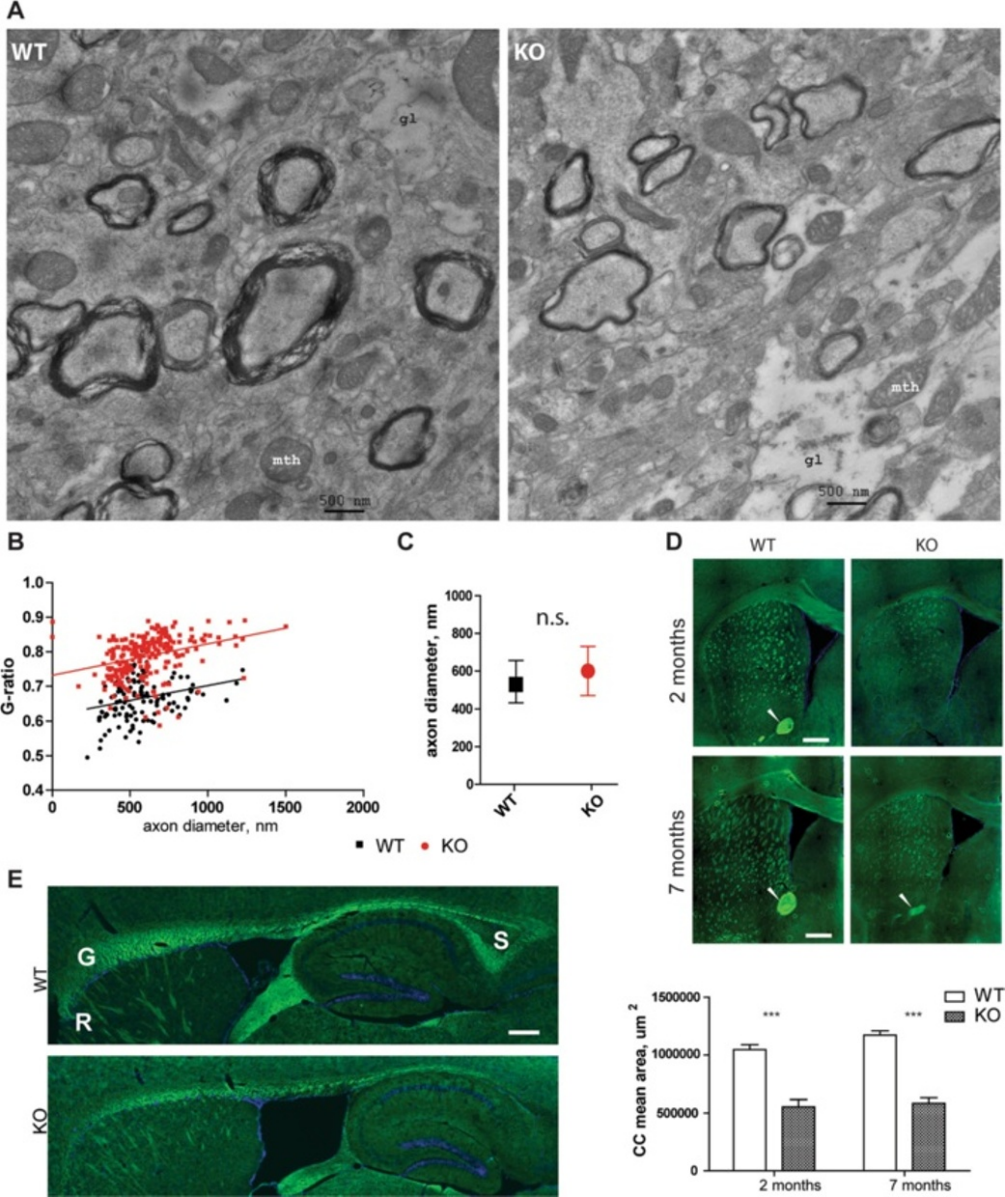
**Myelination is impaired in** ***Mcoln1*** ^***−/−***^ **mice. (A)**. Representative electron micrographs of CA1 stratum radiatum in 7 month-old control (WT) and *Mcoln1* ^*−/−*^ (KO) mice showing reduced thickness of myelin sheaths. **(B)**. G-ratio (axon diameter/fiber diameter) plotted against axon diameter shows reduced degree of myelination in *Mcoln1* ^*−/−*^ (124 axons; 4 mice) compared to wild-type (231 axon; 4 mice) 7 month-old littermates, p (clustered rank-sum test) = 0.018. **(C).** Comparison of axon diameters revealed no significant difference between control (WT) and *Mcoln1* ^*−/−*^ (KO) littermates. Data presented as median values with interquartile range, n.s., p (clustered rank-sum test) = 0.28. **(D)**. Representative images of FluoroMyelin Green- stained coronal sections show reduced myelination of corpus callosum, internal capsule and anterior commissure (indicated by white arrowheads) in *Mcoln1* ^*−/−*^ brain and thinning of the corpus callosum (*n* = 3 per genotype at 2 months, *n* = 5 per genotype at 7 months; p (t-test) <0.01). **(E)**. Representative images of FluoroMyelin Green- stained sagittal sections (counterstained with DAPI) demonstrate malformation of the corpus callosum in the *Mcoln1* ^*−/−*^ at 2 months. S-splenium, G-genu, R-rostrum. Scale bars = 0.5 mm.

The most remarkable ultrastructural feature observed by electron microscopy in the stratum radiatum of *Mcoln1*^*−/−*^ mice was reduced myelination (Figure 5A). Morphometric analysis revealed significant thinning of the myelin sheaths as shown by significantly lower g-ratio (axon diameter/fiber diameter, where fiber diameter is a sum of axon diameter and myelin sheath thickness) in *Mcoln1*^*−/−*^ mice (Figure 5B). No significant difference was observed in the diameter of myelinated axons between the two genotypes (Figure 5C). Impaired CNS myelination and a dysgenic corpus callosum are hallmarks of neuropathology in MLIV patients, and a decreased Luxol fast blue staining has been previously reported in the corpus callosum, deep layers of neocortex and cerebellar white matter tracts of the end-stage *Mcoln1*^*−/−*^ mice [26]. To follow the time-course of myelination deficits, we set out to measure myelination in two and seven month-old *Mcoln1*^*−/−*^ mice using FluoroMyelin Green stain. Analysis of coronal sections (Figure 5D) revealed profound thinning of the corpus callosum and reduced myelination of internal capsule in *Mcoln1*^*−/−*^ mice at both time points. Strikingly, we observed no anterior commissure (AC) in the *Mcoln1*^*−/−*^ mice at two months. However, a dysmorphic and smaller AC appeared in the sections from seven month-old mice (Figure 5D). Analysis of the FlyoroMyelin Green staining in the sagittal sections revealed malformation of the corpus callosum (CC) in the *Mcoln1*^*−/−*^ mice. Specifically, similar to MLIV patients, *Mcoln1*^*−/−*^ mice displayed a generally hypomorphic corpus callosum, with dysgenic rostrum and dysgenic or absent splenium compared to WT littermate controls (Figure 5E).

## Discussion

The most striking clinical manifestation of MLIV is psychomotor retardation resulting in the developmental arrest of motor, speech and cognitive function at the level of about 15 months. Thus, understanding the early pathologic events leading to neurologic and visual impairment is of prime importance for designing an effective therapy for MLIV. To study the first signs of neuropathology and to follow their maturation with disease progression, we performed open field testing on young animals and correlated our findings with systematic volumetric and histopathologic analysis and *in vivo* resting calcium imaging of the *Mcoln1*^*−/−*^ mouse brain. Our results reveal that early motor and cognitive deficits in *Mcoln1*^*−/−*^ mice are associated with white matter abnormalities, including hypogenesis of the corpus callosum, and pronounced activation of microglia and astrocytes, which is unexpectedly not accompanied by neurotoxicity or neuronal loss. However, we observe disrupted synaptic function, and increased length of asymmetric postsynaptic densities in excitatory synapses in the hippocampus of a MLIV mouse model at the late stage of disease.

Given the early onset of psycho-motor disabilities in patients with MLIV [5],[37], we tested spontaneous locomotor and exploratory activity in juvenile one month-old or two month-old young adult mice. Our results revealed that the overall performance in the open field test changes with age, with a significant increase in the wild-type littermates' activity at two months of age compared to one month. However, while we observed no significant differences between the performance of one month-old wild-type and *Mcoln1*^*−/−*^ mice, these mutant mice fail to undergo the normal developmental progression and showed significantly decreased jumping, rearing and exploratory activity at two months compared to their control littermates, indicating an onset of the locomotor and potentially cognitive decline due to the loss of TRPML1 at this age.

A striking early phenotype that we observed in *Mcoln1*^*−/−*^ mice was activation of microglia and astrocytes. Activation of astrocytes has also been reported in the only brain autopsy case described in MLIV [9], where, based on general observations of reactive astrocytes accompanying neuronal death in other neurodegenerative conditions, it was thought to mark neuronal loss. Reactive astrocytes and microgliosis have been observed in mouse models of other LSDs, including the NCLs [27],[38]–[43], mucopolysaccharidosises [44]; Neimann-Pick disease [45] and mucolipidosis II [46]. In many of these models, neuroinflammation develops early and precedes neuronal loss in the gliosis-affected brain regions. However, our data show that in *Mcoln1*^*−/−*^ mice gliosis was not accompanied by neuronal loss even at the late stage of the disease.

To determine if the loss of TRPML1 or the activation of microglia or astrocytes observed in the *Mcoln1*^*−/−*^ brain affects the physiological status of neurons, we measured resting concentrations of Ca^2+^ in neuronal somata and neuropil in the somatosensory cortex using *in vivo* multi-photon microscopy with genetically encoded calcium indicator YC3.60. The ability of neurons to maintain calcium homeostasis is a functional read-out of neuronal "health". Overload of calcium leading to activation of calcineurin-dependent neurodegenerative processes has been demonstrated in the neurites of several mouse models of Alzheimer's disease [28]. Moreover, in MLIV, intracellular calcium dyshomeostasis due to loss of TRPML1, a lysosomal cation channel permeable to Ca^2+^, has been suggested in the literature as a primary mechanism of the disease [47],[48]. Surprisingly, we observed no changes in resting Ca^2+^ concentration and virtually no effect of cytosolic storage material on intracellular [Ca^2+^] in *Mcoln1*^*−/−*^ neurons of somatosensory cortex at the age of three months, implying spared physiologic status of neurons and absence of excitotoxicity in spite of TRPML1 loss and widespread reactive gliosis in the brain at this age.

Interestingly, in the hippocampus, activation of microglia, which did not cause or result in the loss of CA1 or CA3 neurons, was accompanied by electrophysiological alterations (enhanced PPT and LTP) in the Schaeffer collaterals of the *Mcoln1*^*−/−*^ mice. Of particular interest is the fact that re-expression of *trpml* in glial cells in the Drosophila model of MLIV was sufficient to restore survival, motor function and synaptic transmission [21]. This fact, together with the recent finding that activated microglia can facilitate the induction of LTP by releasing TNF [49], further highlights the role that glial cells may have in MLIV pathogenesis as well in other LSDs; a role which to date has been underappreciated. The mechanism by which loss of TRPML1 causes a glial response is not clear. Recently TRPML1 was shown to be involved in the regulation of phagocytosis in macrophages [50] further suggesting that neuroinflammation in MLIV could be caused by disturbed phagocytic activity in microglia. A more detailed study of the time course and mechanisms of neuroinflammation in *Mcoln1*^*−/−*^ mice and primary cultures of microglia and astrocytes will be in the focus of our future work.

Very little is known about brain pathology in human MLIV due to the lack of systematic clinical studies. An MRI study of 15 patients with MLIV revealed a characteristic developmental impairment of the corpus callosum, white matter abnormalities on T1-weighed images and deposition of ferritin in basal ganglia and thalamus reflected by the changes in the signal intensities on T1- and T2- weighted MRIs [8]. In all but one MLIV patient with mild clinical manifestations, the corpus callosum was uniformly thinned, and had no rostrum together with an absent or dysplastic splenium. The thickness of corpus callosum in the patients with typical MLIV ranging from 16 months to 22 years of age varied from 2 to 3 mm which corresponds to the normal thickness at one month of age. These findings have been confirmed in the recent study recruiting five more MLIV patients of ages from 7 to 18 year-old [6]. Interestingly, we observed similar malformation of the corpus callosum with hypogenesis of the genu and trunk and characteristic agenesis of the rostrum and splenium in two month-old *Mcoln1*^*−/−*^ mice. Developmental defects of the corpus callosum can be caused by impaired cell proliferation and migration, gliogenesis, axon growth, guidance or myelination. The mechanism resulting in dysgenic corpus callosum due to the loss of TRPML1 remains unclear. However, our data showing preserved cortical volume, thickness and neuron numbers in *Mcoln1*^*−/−*^ mice make deficits in cell proliferation or neuron loss unlikely. Interestingly, a hypoplastic corpus callosum and other white matter abnormalities are found in many other LSDs: Gaucher, Krabbe, Pompe, Niemann-Pick, NCLs, mannosidosises, gangliosidoses and mucopolysaccharosidosises (reviewed in [51]). These lysosomal diseases have an early onset in the first two years of life, a period critical for myelination, implying the role of aberrant lipid metabolism in oligodendrocytes in the pathogenesis of such disorders. Our data showing early myelination abnormalities in corpus callosum and other white matter structures such us internal capsule and anterior commissure and reduced thickness of myelin sheaths in the stratum radiatum of *Mcoln1*^*−/−*^ mice further support this idea.

Our electrophysiological data showing enhanced PPF in the Schaefer collateral pathway are consistent with the previous report of decreased synaptic transmission in neuromuscular junctions in the Drosophila model of MLIV, which was shown to arise from the presynaptic impairment of synaptic vesicle cycling [21]. Notably, our results indicated a trend toward decreased synaptic transmission and decreased synapse density in MLIV mice, although these differences did not reach statistical significance. The significant increase in PSD length that we observed at excitatory synapses of CA1 neurons may represent a compensatory increase in postsynaptic active zone size in response to reduced presynaptic input, or impaired internalization of AMPA-type glutamate receptors. Our data also raise the possibility that reduced myelination, the most remarkable ultrastructural finding, may contribute to the decreased presynaptic function we observed in MLIV mice. We suggest that the thinning of myelin sheaths in Schaeffer collaterals may affect the efficiency of glutamate release through impaired propagation of action potentials and, thus, contribute to the development of cognitive impairment in MLIV. Massive axonal degeneration and formation of numerous axonal spheroids, which are full of aggregated mitochondria and vesicular electron-dense structures, has been previously shown in cerebellar Purkinje neurons in the end stage *Mcoln1*^*−/−*^ mice, indicating defects in axonal transport [26]. In contrast, we observed no such structures and no changes in the axon caliber in Schaeffer collaterals, suggesting that pathway dependent axonal pathology occurs in these mice.

Overall, our data reveals that the glial activation is a dramatic and early feature of MLIV, and it is not associated with overt neurodegeneration in the course of disease. This finding changes our understanding of the disease mechanisms, shifting focus towards the role of lysosomes in glial cell health and functioning, and the role of glial dysfunction in the etiology of MLIV and other lysosomal diseases. It also opens new frontiers in therapy development to prevent or reverse the devastating neurologic symptoms of this disease. More specifically, along with traditional therapeutic approaches for LSDs involving substrate reduction or gene therapy, new paradigms such as glial progenitor cell replacement therapy or modulation of neuroinflammation can also be considered in MLIV.

## Authors' information

Andrew M.S. Wong, Jonathan D. Cooper and Susan A. Slaugenhaupt co-senior authors.

## Additional files

## Electronic supplementary material

Additional file 1: Figure S1.: Brain atrophy is absent in *Mcoln1* ^*−/−*^ mice in the course of disease. (A). Cavalieri estimates of the volume of hippocampus, cortex, thalamus and striatum obtained from wild-type (WT) and *Mcoln1* ^−/−^ (KO) littermates at two (*n* = 6 per genotype), three (*n* (WT) = 3; *n* (KO) = 4) and seven months of age (*n* = 4 per genotype). (B). Cortical thickness measured in the somatosensory barrelfield (S1BF), primary motor (M1), lateral entorhinal (LEnt) and primary visual (V1) areas. Two-way ANOVA (genotype x age) shows no significant interaction or significant effects of genotype at any of examined brain regions. (TIFF 3 MB)

Additional file 2: Figure S2.: Iba1 staining confirms microglia activation in *Mcoln1* ^*−/−*^ mice. Representative images of Iba1 immunostaning in wild-type (WT) and *Mcoln1* ^−/−^ (KO) littermates at two months of age showing increased immunoreactivity in KO in somatosensory barrelfield cortex. Scale bar is equal to 150 μm. Sections were counterstained with Gill Hematoxylin. (TIFF 3 MB)

Additional file 3: Figure S3.: Intra-glial storage in CA1 stratum radiatum of *Mcoln1* ^*−/−*^ mice. (A). Representative electron micrograph showing accumulation of characteristic MLIV storage bodies with electron-dense granular and lamellar material (white arrowhead) in an astrocyte. (B). Accumulation of clusters of lysosome-like storage inclusions in a microglial cell or macrophage. (TIFF 8 MB)

Additional file 4: Figure S4.: Absence of neuronal loss in *Mcoln1* ^*−/−*^ mice. Optical fractionator estimates of number of neurons in the VPL-VPM, DLG and red nucleus of the thalamus, S1BF, and CA1 and CA3 subfields of the hippocampus in 7 month-old wild-type (WT) and *Mcoln1* ^−/−^ (KO) littermates (*n* = 4 per genotype). Data analyzed by t-test and show no significant differences between WT and KO in any of the examined brain regions. (TIFF 11 MB)

Below are the links to the authors’ original submitted files for images.

Authors’ original file for figure 1

Authors’ original file for figure 2

Authors’ original file for figure 3

Authors’ original file for figure 4

Authors’ original file for figure 5
