# Supplementary figures and images for: Behavioral deficits, early gliosis, dysmyelination and synaptic dysfunction in a mouse model of mucolipidosis IV
Source: Acta Neuropathol Commun. 2014 Sep 9;2:133. doi: 10.1186/s40478-014-0133-7 (PMC4173007; doi:10.1186/s40478-014-0133-7)

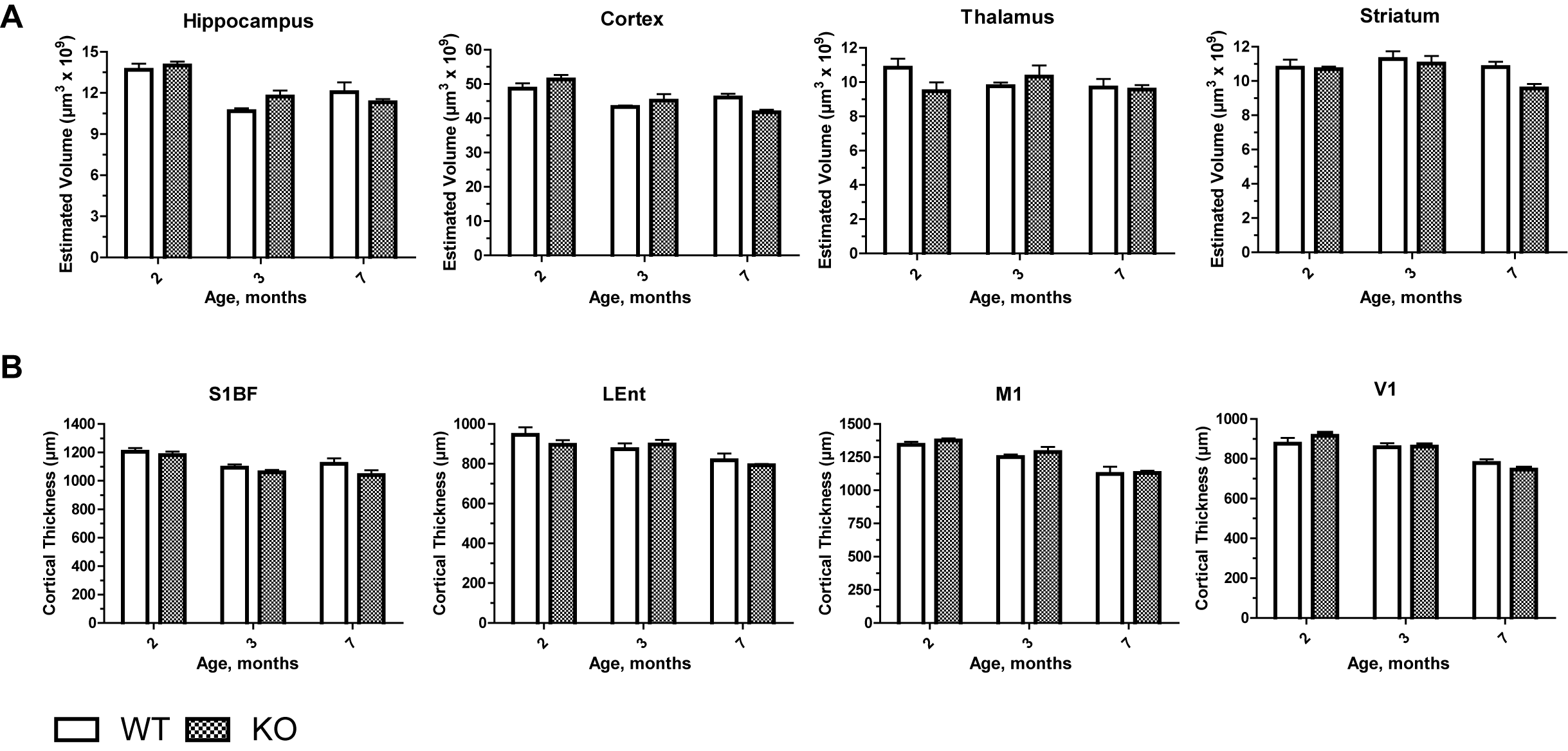

Supplement: Supplementary file 1 — Additional file 1: Figure S1.: Brain atrophy is absent in Mcoln1 −/− mice in the course of disease. (A). Cavalieri estimates of the volume of hippocampus, cortex, thalamus and striatum obtained from wild-type (WT) and Mcoln1 −/− (KO) littermates at two (n = 6 per genotype), three (n (WT) = 3; n (KO) = 4) and seven months of age (n = 4 per genotype). (B). Cortical thickness measured in the somatosensory barrelfield (S1BF), primary motor (M1), lateral entorhinal (LEnt) and primary visual (V1) areas. Two-way ANOVA (genotype x age) shows no significant interaction or significant effects of genotype at any of examined brain regions. (TIFF 3 MB) [file 40478_2014_9133_MOESM1_ESM.tiff]

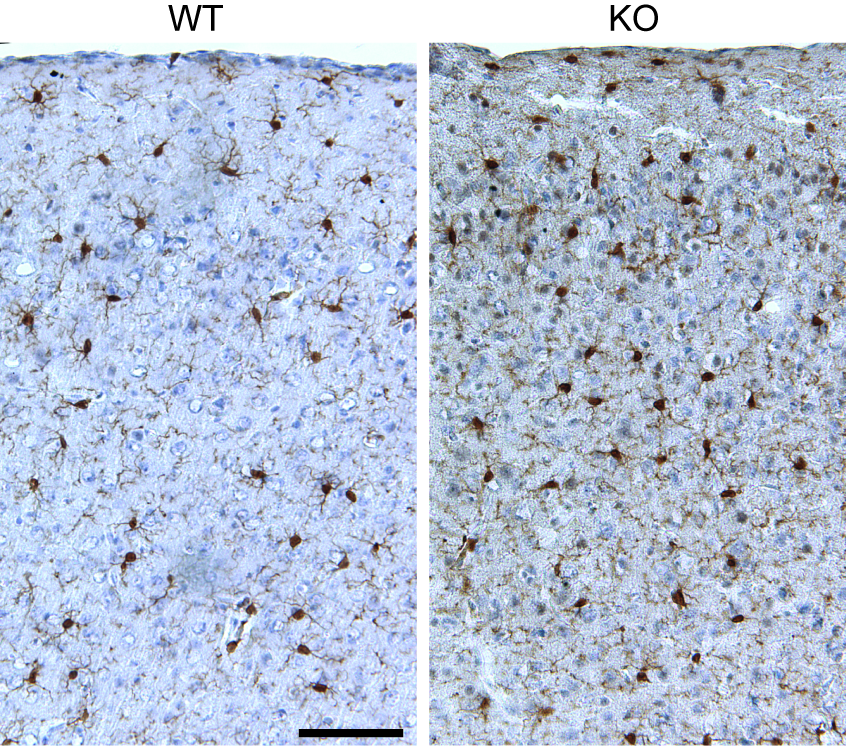

Supplement: Supplementary file 2 — Additional file 2: Figure S2.: Iba1 staining confirms microglia activation in Mcoln1 −/− mice. Representative images of Iba1 immunostaning in wild-type (WT) and Mcoln1 −/− (KO) littermates at two months of age showing increased immunoreactivity in KO in somatosensory barrelfield cortex. Scale bar is equal to 150 μm. Sections were counterstained with Gill Hematoxylin. (TIFF 3 MB) [file 40478_2014_9133_MOESM2_ESM.tiff]

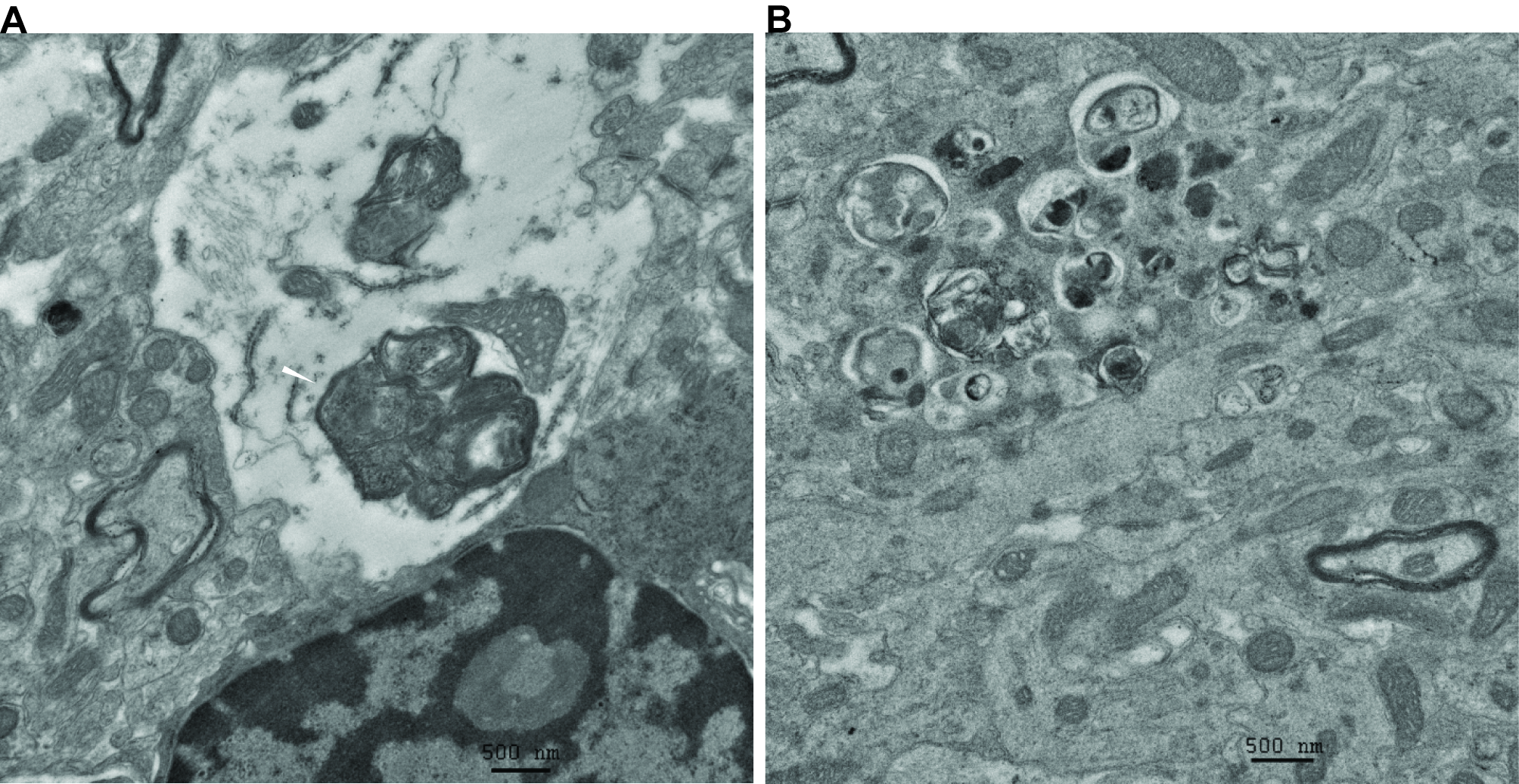

Supplement: Supplementary file 3 — Additional file 3: Figure S3.: Intra-glial storage in CA1 stratum radiatum of Mcoln1 −/− mice. (A). Representative electron micrograph showing accumulation of characteristic MLIV storage bodies with electron-dense granular and lamellar material (white arrowhead) in an astrocyte. (B). Accumulation of clusters of lysosome-like storage inclusions in a microglial cell or macrophage. (TIFF 8 MB) [file 40478_2014_9133_MOESM3_ESM.tiff]

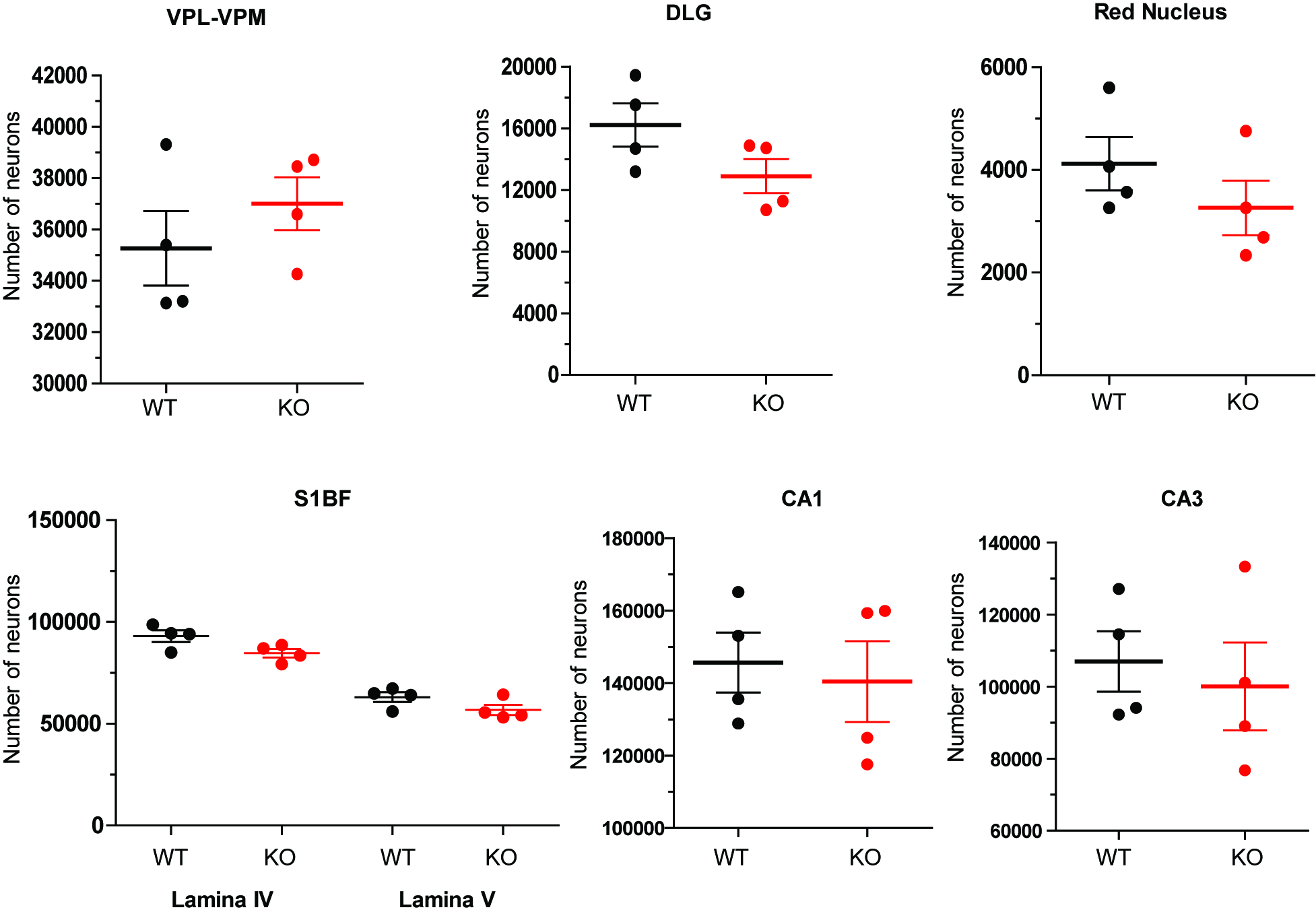

Supplement: Supplementary file 4 — Additional file 4: Figure S4.: Absence of neuronal loss in Mcoln1 −/− mice. Optical fractionator estimates of number of neurons in the VPL-VPM, DLG and red nucleus of the thalamus, S1BF, and CA1 and CA3 subfields of the hippocampus in 7 month-old wild-type (WT) and Mcoln1 −/− (KO) littermates (n = 4 per genotype). Data analyzed by t-test and show no significant differences between WT and KO in any of the examined brain regions. (TIFF 11 MB) [file 40478_2014_9133_MOESM4_ESM.tiff]

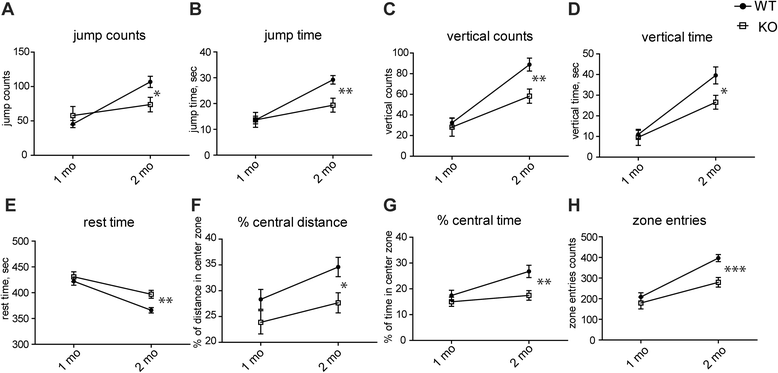

Supplement: Supplementary file 5 — Authors’ original file for figure 1 [file 40478_2014_9133_MOESM5_ESM.gif]

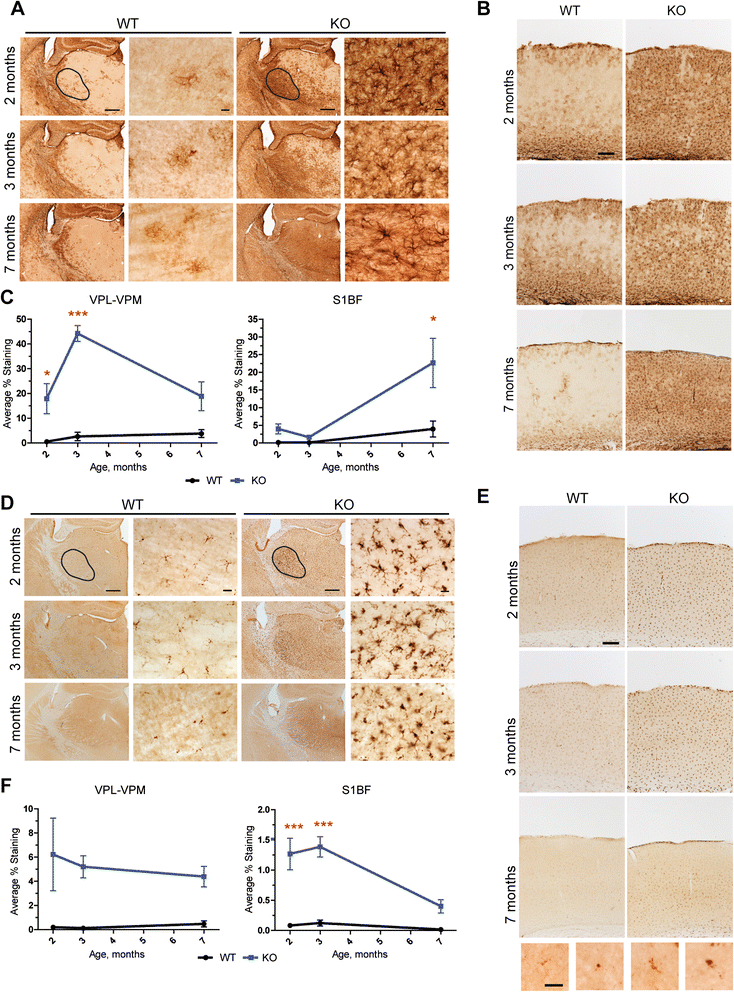

Supplement: Supplementary file 6 — Authors’ original file for figure 2 [file 40478_2014_9133_MOESM6_ESM.gif]

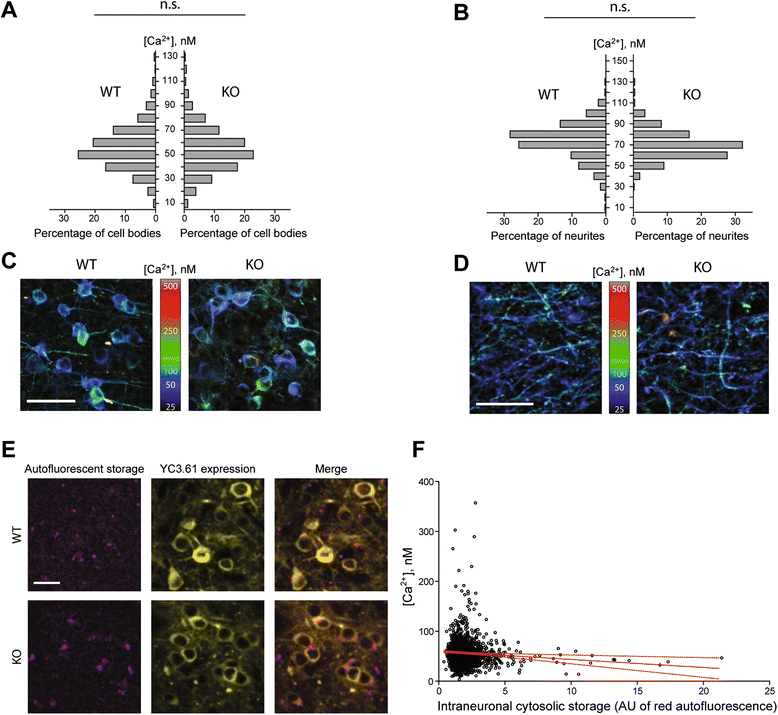

Supplement: Supplementary file 7 — Authors’ original file for figure 3 [file 40478_2014_9133_MOESM7_ESM.gif]

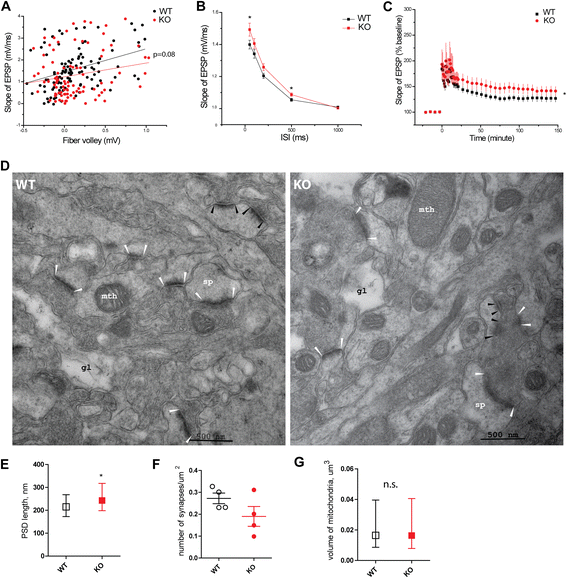

Supplement: Supplementary file 8 — Authors’ original file for figure 4 [file 40478_2014_9133_MOESM8_ESM.gif]

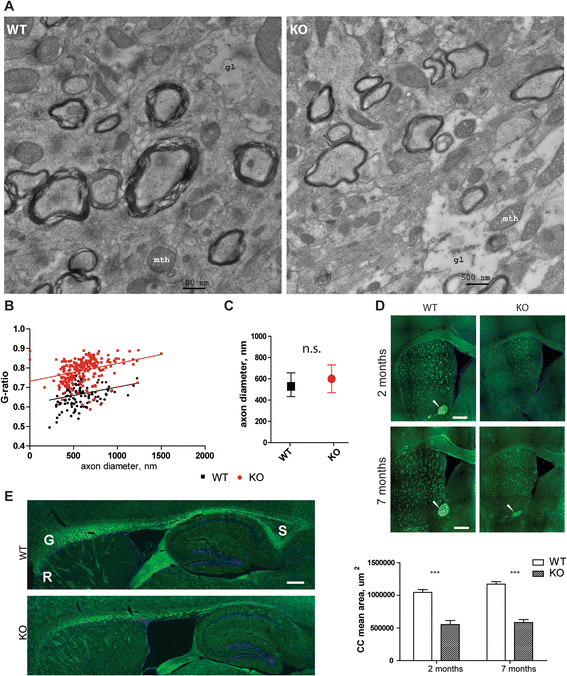

Supplement: Supplementary file 9 — Authors’ original file for figure 5 [file 40478_2014_9133_MOESM9_ESM.gif]
